# A Model for the Evolution of Nucleotide Polymerase Directionality

**DOI:** 10.1371/journal.pone.0018881

**Published:** 2011-04-22

**Authors:** Joshua Ballanco, Marc L. Mansfield

**Affiliations:** 1 Department of Chemistry, Chemical Biology, and Biomedical Engineering, Stevens Institute of Technology, Hoboken, New Jersey, United States of America; 2 Energy Dynamics Laboratory, Bingham Research Center, Vernal, Utah, United States of America; National University of Ireland Galway, Ireland

## Abstract

**Background:**

In all known living organisms, every enzyme that synthesizes nucleic acid polymers does so by adding nucleotide 5′-triphosphates to the 3′-hydroxyl group of the growing chain. This results in the well known 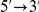 directionality of all DNA and RNA Polymerases. The lack of any alternative mechanism, e.g. addition in a 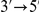 direction, may indicate a very early founder effect in the evolution of life, or it may be the result of a selective pressure against such an alternative.

**Methodology/Principal Findings:**

In an attempt to determine whether the lack of an alternative polymerase directionality is the result of a founder effect or evolutionary selection, we have constructed a basic model of early polymerase evolution. This model is informed by the essential chemical properties of the nucleotide polymerization reaction. With this model, we are able to simulate the growth of organisms with polymerases that synthesize either 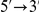 or 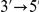 in isolation or in competition with each other.

**Conclusions/Significance:**

We have found that a competition between organisms with 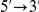 polymerases and 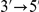 polymerases only results in a evolutionarily stable strategy under certain conditions. Furthermore, we have found that mutations lead to a much clearer delineation between conditions that lead to a stable coexistence of these populations and conditions which ultimately lead to success for the 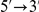 form. In addition to presenting a plausible explanation for the uniqueness of enzymatic polymerization reactions, we hope these results also provide an example of how whole organism evolution can be understood based on molecular details.

## Introduction

An oft cited piece of evidence for the common origin of life is the observation that all living organisms use similar enzymes which synthesize nucleotide polymers in the same, 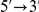, direction to produce copies of their genetic information. The problem with this argument is that it presupposes that this universal similarity can only result from a continuous line of descent from a single common progenitor. One might reasonably wonder if a reversed, 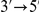, polymerase could have existed at some point in the past and, subsequently, question why such a polymerase is not currently found anywhere in nature.

Approaching this problem, it is easy enough to imagine a polymerase which operates in much the same fashion as modern nucleotide polymerases, but uses as its substrate nucleotides with 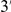-triphosphate moieties in place of nucleotide-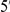-phosphates. This hypothetical situation can be discounted, however, by considering that ribose-3-phosphate is known to decompose more readily under mildly acidic conditions than ribose-5-phosphate [1], consistent with the availability of a nucleophilic oxygen on the adjacent carbon at position 2 in ribose-3-phosphate. The implication is that the primordial pool of nucleotides would lack sufficient quantities of nucleotide-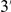-triphosphates with which to do synthesis. So, we can assume that any reverse polymerase would have to work with the same nucleotide-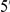-triphosphates as a 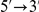 polymerase, which it could do by adding new nucleotides to an activated 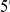 end of the growing nucleotide chain.

So the question remains: why do we not currently observe such polymerases in nature? There are three explanations one might imagine for why no alternative polymerases are found in nature: chemical impossibility, founder effect, or evolutionary selection. Taking the first explanation, it may be that the chemistry involved in synthesis in the reverse direction, 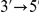, is impossible, and only the known 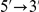 polymerization reaction can be performed by biological enzymes. A cursory look at the active site mechanism of known nucleotide polymerases makes it trivial to reject this possibility. Polymerization in DNA and RNA polymerase enzymes occurs via a dehydration reaction that joins the hydroxyl group on the phosphate of a nucleotide triphosphate and the hydroxyl group of the terminal monomer on the growing nucleic acid chain [2]. In this mechanism two divalent metal cations, coordinated by a number of acidic amino acids, facilitate the transfer of an electron pair from the free 3′ hydroxyl group to the 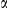-phosphate [3]. What is notable about this mechanism is that catalysis does not involve segments of the growing polymer or the nucleotide monomer other than the 3′ hydroxyl and 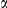-phosphate. That is, if the triphosphate group were attached to the growing chain instead, and the 3′ hydroxyl group were positioned on the incoming nucleotide monomer, the active site configuration would not look any different. It is also notable that the change in entropy of the reaction would be the same regardless of polymerization reaction, as the leaving group is a pyrophosphate in either case.

Taking the second explanation, it is possible that both forms of nucleotide polymerase existed during the early evolution of life. If the bulk of these proto-lifeforms were exterminated, leaving only a small sub-population to continue growing and reproducing, eventually leading to all current forms of life, and if this small sub-population contained organisms with exclusively 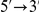 nucleotide polymerases, then we would expect all life to contain 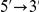 polymerases regardless of the fitness of this or any alternative form. This is an example of a founder effect [4].

Finally, the last possible explanation is that polymerizing nucleotides in a 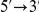 direction confers some advantage which may be selected for. It is this possibility with which we are primarily concerned. To understand how polymerization in one direction might be able to impart an advantage on an organism, we should look a bit closer at the chemistry of nucleotides and nucleic acid synthesis. One very important aspect to consider is the fact that nucleotide triphosphates in an aqueous solution will spontaneously hydrolyze with first-order reaction kinetics [5]. A full triphosphate group is required for successful joining of a nucleotide to a growing nucleic acid chain, so this sort of spontaneous hydrolysis represents a block to further synthesis. In the case of a 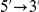 polymerization reaction, the reactive triphosphate is on the incoming nucleotide and so a spontaneous loss of the triphosphate can be compensated for by finding a new nucleotide substrate. Contrast this to the case of a 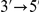 polymerization reaction, where the reactive triphosphate is located on the growing chain. A spontaneous loss of the triphosphate from the growing chain would require either disposing of the entire polymerization product or employing a secondary enzyme activity to replace the active triphosphate group. Therefore, the penalty to the polymerization rate on spontaneous hydrolysis of a triphosphate is greater for the 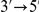 polymerizing direction than for the 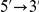 direction.

However, not all nucleotide polymerases operate at the same rate, and a 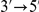 polymerase could compensate for the spontaneous triphosphate hydrolysis penalty by evolving a faster synthetic rate. Since spontaneous hydrolysis of the triphosphate group occurs with a fixed rate constant at a constant temperature, a faster polymerase will be able to add more nucleotides to a growing chain between each such hydrolysis event, reducing the aggregate penalty for a given length of nucleic acid. Such an evolutionary path is not without consequence, though, as the speed of polymerase synthesis is tied to mutation rate [6]. The question we are interested in is whether the combination of the increased penalty for spontaneous triphosphate hydrolysis and the inability to evolve a faster polymerase without also incurring an increased mutation rate is enough to explain the absence of 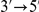 nucleotide polymerases in nature by natural selection alone. The model we present here was constructed to address this question directly.

## Results

### Simulating Polymerase Evolution

We constructed a model system that consisted of an environment at a certain temperature containing a number of model organisms each with a genome and a polymerase. The environment was constrained with a fixed maximum population and was designed so that individual organisms would be randomly culled with a frequency proportional to the inverse of the remaining capacity in the environment. This culling was designed to mimic observations of density dependent growth inhibition of bacterial cultures [7]. Each organism was modeled as a state machine that was in either a replicating or dividing state. In the replicating state, the organism's polymerase adds nucleotides to a new copy of the organism's genome until the copy is complete. At this point, the organism shifts to a dividing state. In this state, the organism attempts to divide and add a new individual to the environment. If the environment is at capacity, the the organism remains in the dividing state. If division is successful, the new organism and the original organism both return to the replicating state.

Each polymerase was set with an intrinsic replication rate and a directionality upon virtual translation from the daughter organism's genome following a replication event. This rate controls the number of nucleotides that a polymerase can add to a new genome during each round of the simulation, and was allowed to vary up to 10-fold based on rules set out in the model. Polymerases incorporated incorrect nucleotides with an error rate determined by both their speed and the temperature of the simulation. Alternatively, the model could be set to disallow mutation, in effect reducing the polymerase error rate to zero.

During each simulation time step, each polymerase was allowed to add a number of nucleotides equal to or less than its rate, but at each addition there was a random chance that the nucleotide substrate would experience a spontaneous hydrolysis of its triphosphate group. In the case that the polymerase was of the 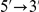 (forward) variety, such an event resulted in a missing incorporation event. In the case that the polymerase was of the 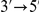 (reverse) variety, a spontaneous hydrolysis event would result in premature termination of that round of additions. The goal with this premature termination was to capture, in the model, the time it would take to repair the loss of the triphosphate group on the growing 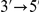 strand. One could imagine that this repair could take the form of a separate enzyme or a secondary function of the polymerase itself. By terminating the current round of addition, we were able to simply model this repair process in a very generic fashion. In the future, it may be interesting to further investigate the effect that different specific repair kinetics might have on the modeled evolution.

The probability of a hydrolysis event occurring was modeled as a Boltzmann distribution. Because the probability of a hydrolysis event also depends on time, the Boltzmann distribution was scaled inversely with polymerase rate for the reverse polymerases. Such scaling is not required for the forward polymerases as it was assumed that the probability of a hydrolysis event in this case was averaged over the available pool of nucleotide triphosphates in a time independent manner.

The genome of each organism was set to a given size at the start of a simulation. Each genome stored information about the rate and directionality of the polymerase for which it coded. This information was used to produce a model polymerase during virtual translation. During the simulation, the number of errors introduce while replicating each genome was tracked, and this number was used to determine by how much the polymerase rate coded for in each daughter genome should differ from that of its mother genome. The directionality of a polymerase was not allowed to change during simulation. For every experiment performed with this model, 1000 was used as the maximum population size and genome size. Polymerase rates were allowed to vary between values of 1 and 10.

### Evolution During Exponential Growth

**Figure 1:**
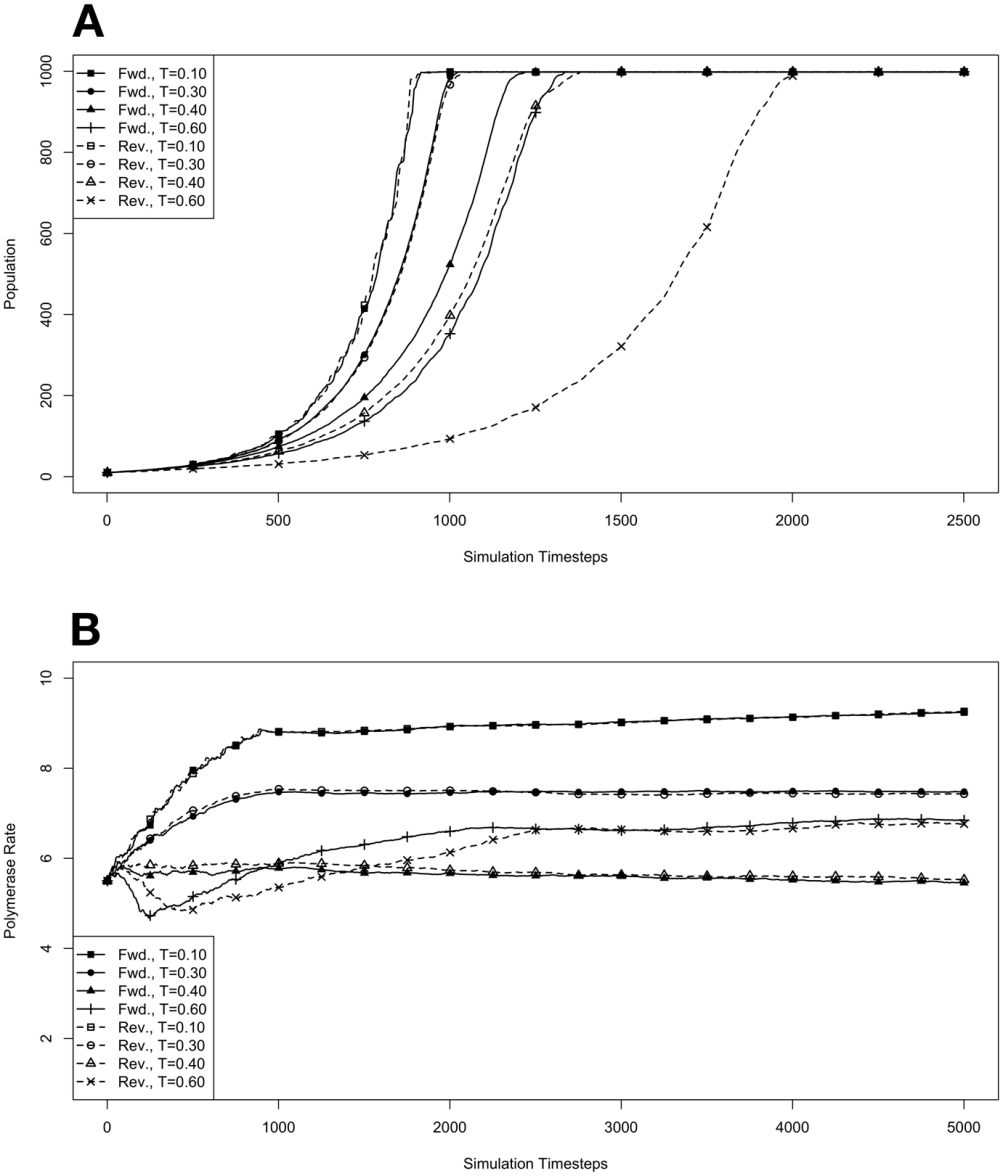
Exponential growth at various temperatures in the absence of competition, with mutations. The model system was seeded with environments, at simulation temperatures of 0.10, 0.30, 0.40, or 0.60, containing 10 organisms with a 5.5 average polymerase rate. **A**. Population size of model organisms as a function of simulation time. **B**. Evolution of the average polymerase rate for the organisms in each environment as a function of simulation time. In each case, solid lines are used to indicate environments with forward polymerizing organisms and dashed lines are for reverse polymerizing organisms. Different temperatures are indicated with different data markers as indicated in the figure legend, and are expressed in units of 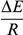.

**Figure 2:**
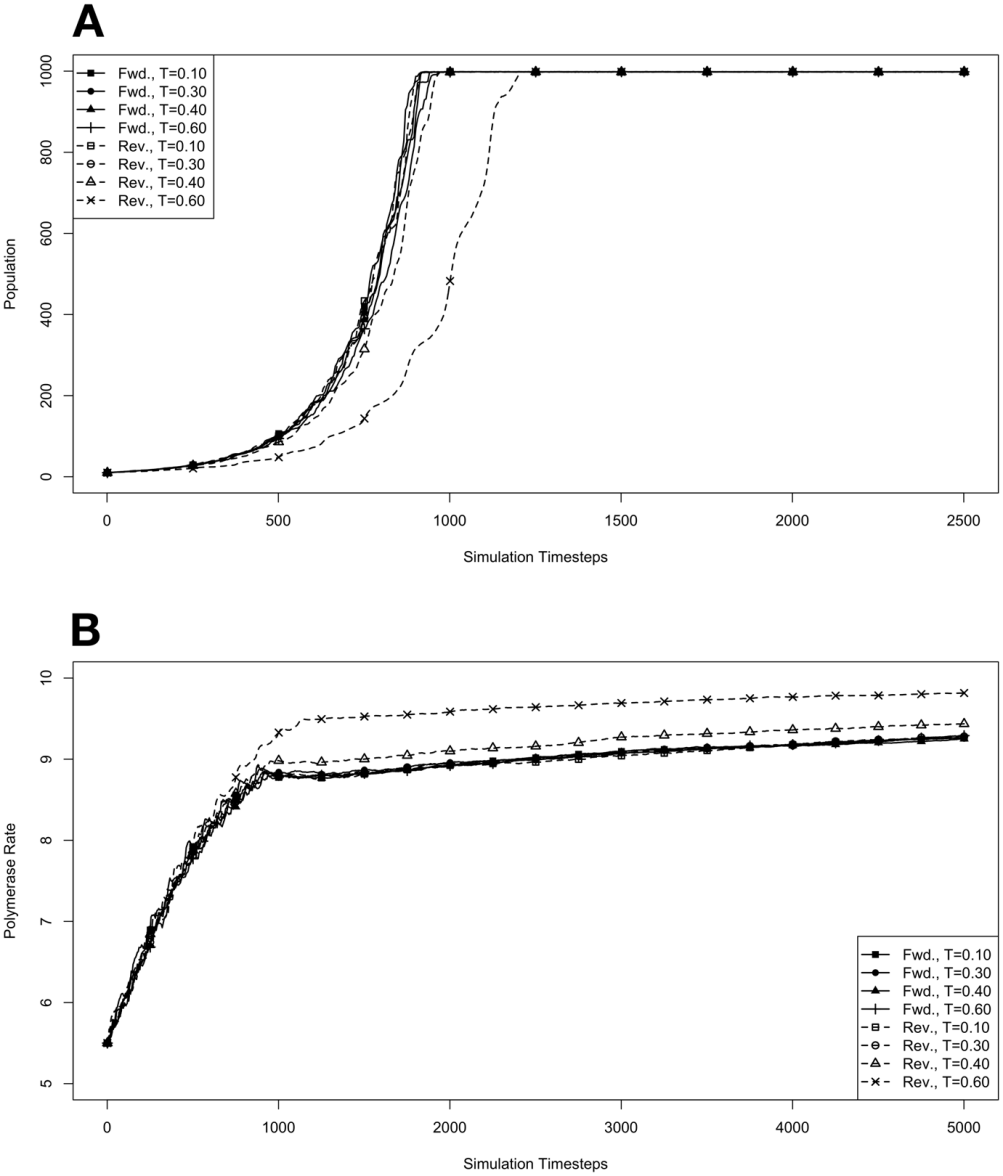
Exponential growth at various temperatures in the absence of competition, with no mutations. The model system was seeded with environments, at simulation temperatures of 0.10, 0.30, 0.40, or 0.60, containing 10 organisms with a 5.5 average polymerase rate. **A**. Population size of model organisms as a function of simulation time. **B**. Evolution of the average polymerase rate for the organisms in each environment as a function of simulation time. In each case, solid lines are used to indicate environments with forward polymerizing organisms and dashed lines are for reverse polymerizing organisms. For every simulation mutations were disallowed. Different temperatures are indicated with different data markers as indicated in the figure legend, and are expressed in units of 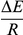.

We began by investigating how organisms with forward or reverse polymerases would behave in the model system during an exponential growth phase in the absence of competitive pressure. To do this, environments at various temperatures were seeded with populations of 10 organisms containing forward or reverse polymerases with all possible values for polymerase rate (yielding an average starting polymerase rate of 5.5). The results of these simulations are presented in figure 1. Simulations were carried out for each type of polymerase individually so that only the dynamics of the polymerase would influence growth. The simulations were also run disallowing mutations. Under this condition there can be no change in polymerase rate for an individual and its daughters from one generation to the next. These results are presented in figure 2. We monitored both the population rate and the average polymerase rate for all the organisms in the environment.

**Figure 3:**
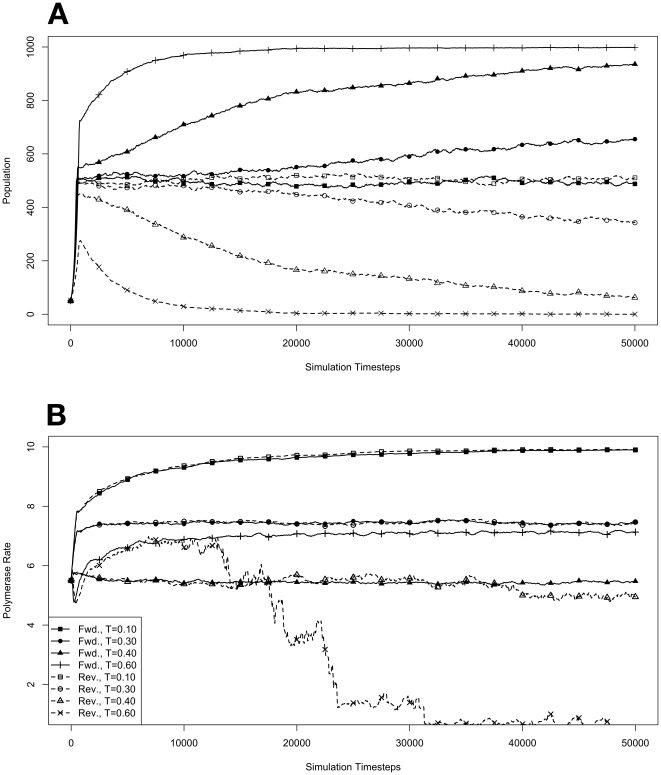
Competition during exponential growth at various temperatures, with mutations. The model system was seeded with environments, at simulation temperatures of 0.10, 0.30, 0.40, or 0.60, containing 100 organisms, 50 each with forward and reverse polymerases, with a 5.5 average polymerase rate. **A**. Population size of model organisms as a function of simulation time. **B**. Evolution of the average polymerase rate for the organisms in each environment as a function of simulation time. In each case, solid lines are used to indicate environments with forward polymerizing organisms and dashed lines are for reverse polymerizing organisms. Different temperatures are indicated with different data markers as indicated in the figure legend, and are expressed in units of 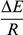.

**Figure 4:**
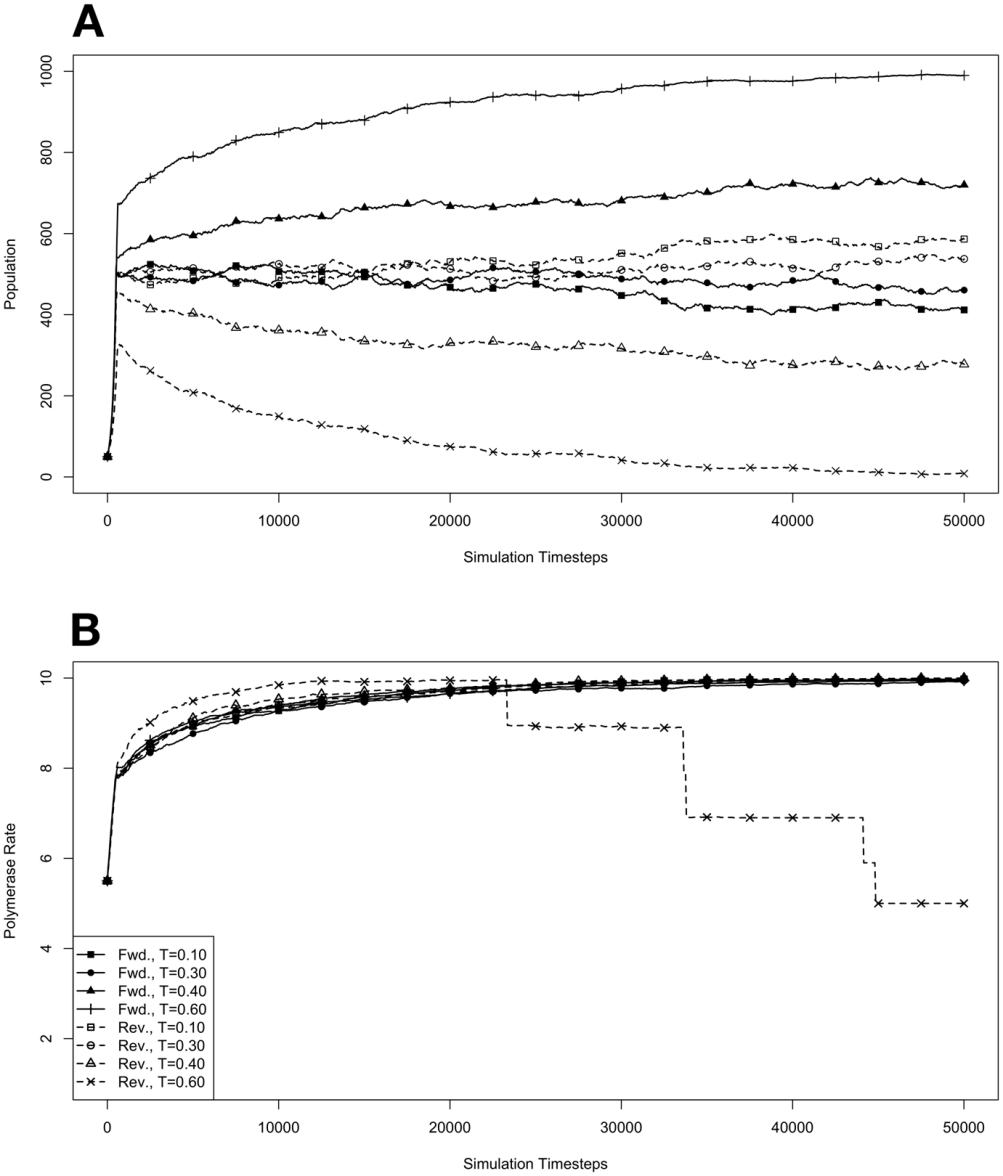
Competition during exponential growth at various temperatures, with no mutations. The model system was seeded with environments, at simulation temperatures of 0.10, 0.30, 0.40, or 0.60, containing 100 organisms, 50 each with forward and reverse polymerases, with a 5.5 average polymerase rate. **A**. Population size of model organisms as a function of simulation time. **B**. Evolution of the average polymerase rate for the organisms in each environment as a function of simulation time. In each case, solid lines are used to indicate environments with forward polymerizing organisms and dashed lines are for reverse polymerizing organisms. For every simulation mutations were disallowed. Different temperatures are indicated with different data markers as indicated in the figure legend, and are expressed in units of 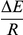.

We then investigated how organisms with forward or reverse polymerases would behave when competing with each other for resources during an exponential growth phase. To do this, we seeded environments with 100 organisms, where 50 of the organisms contained forward polymerases and 50 reverse. For each type, there were 5 organisms with each possible polymerase rates for an average starting polymerase rate of 5.5. The results of these simulations are presented in figure 3. Simulations were again replicated disallowing mutations. These results are presented in figure 4. In each environment, we tracked the subpopulations of organisms with forward and reverse polymerases separately so that we could follow the population changes and polymerase rate evolution for each condition in the mixed case independently.

### Competition in a Full Environment

**Figure 5:**
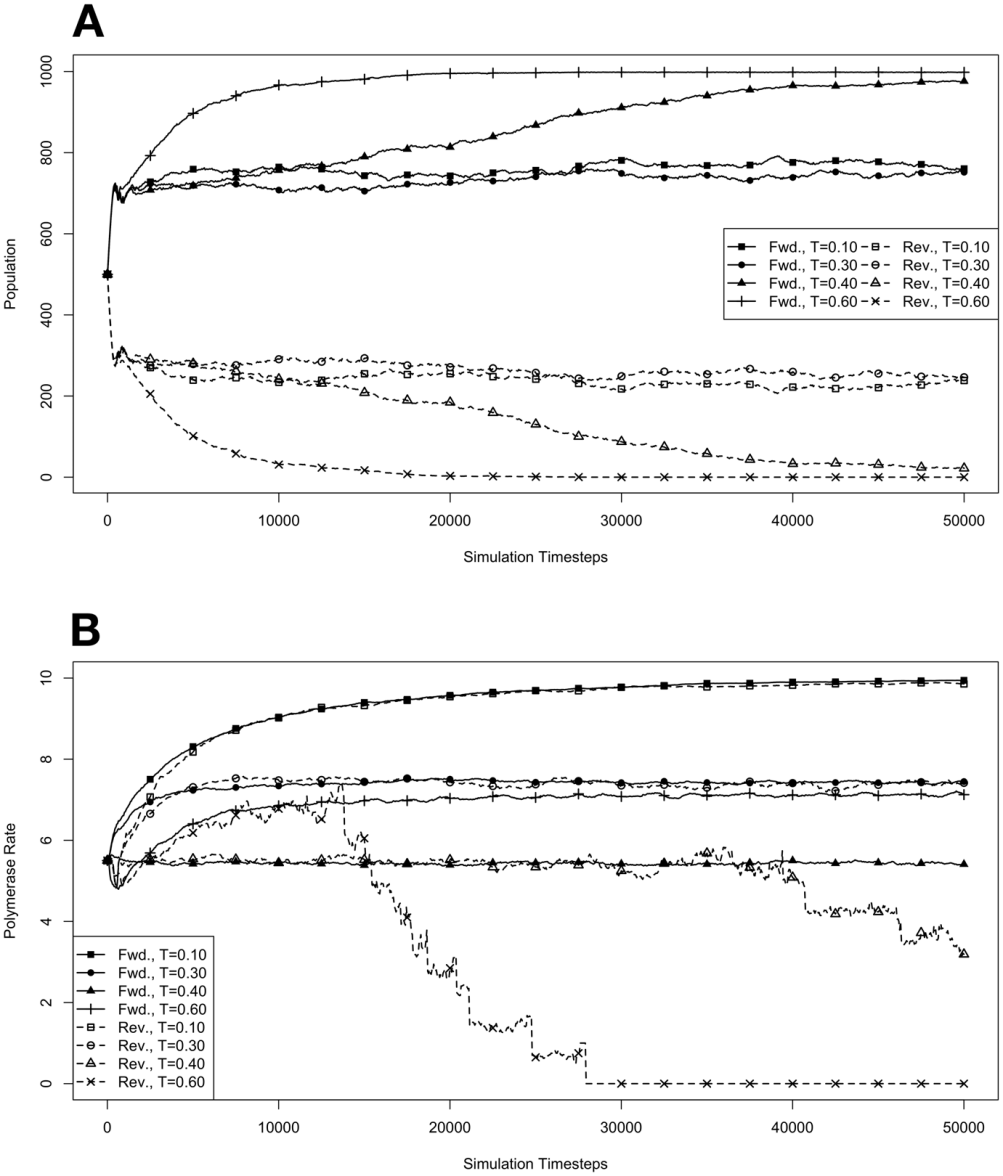
Competition in an environment at maximum capacity, with mutations. Environments, at simulation temperatures of 0.10, 0.30, 0.40, or 0.60, were seeded with 500 organisms containing forward polymerases and 500 containing reverse, both with a 5.5 average polymerase rate. **A**. Population size of model organisms as a function of simulation time. **B**. Evolution of the average polymerase rate for the organisms in each environment as a function of simulation time. In each case, solid lines are used to indicate environments with forward polymerizing organisms and dashed lines are for reverse polymerizing organisms. Different temperatures are indicated with different data markers as indicated in the figure legend, and are expressed in units of 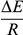.

**Figure 6:**
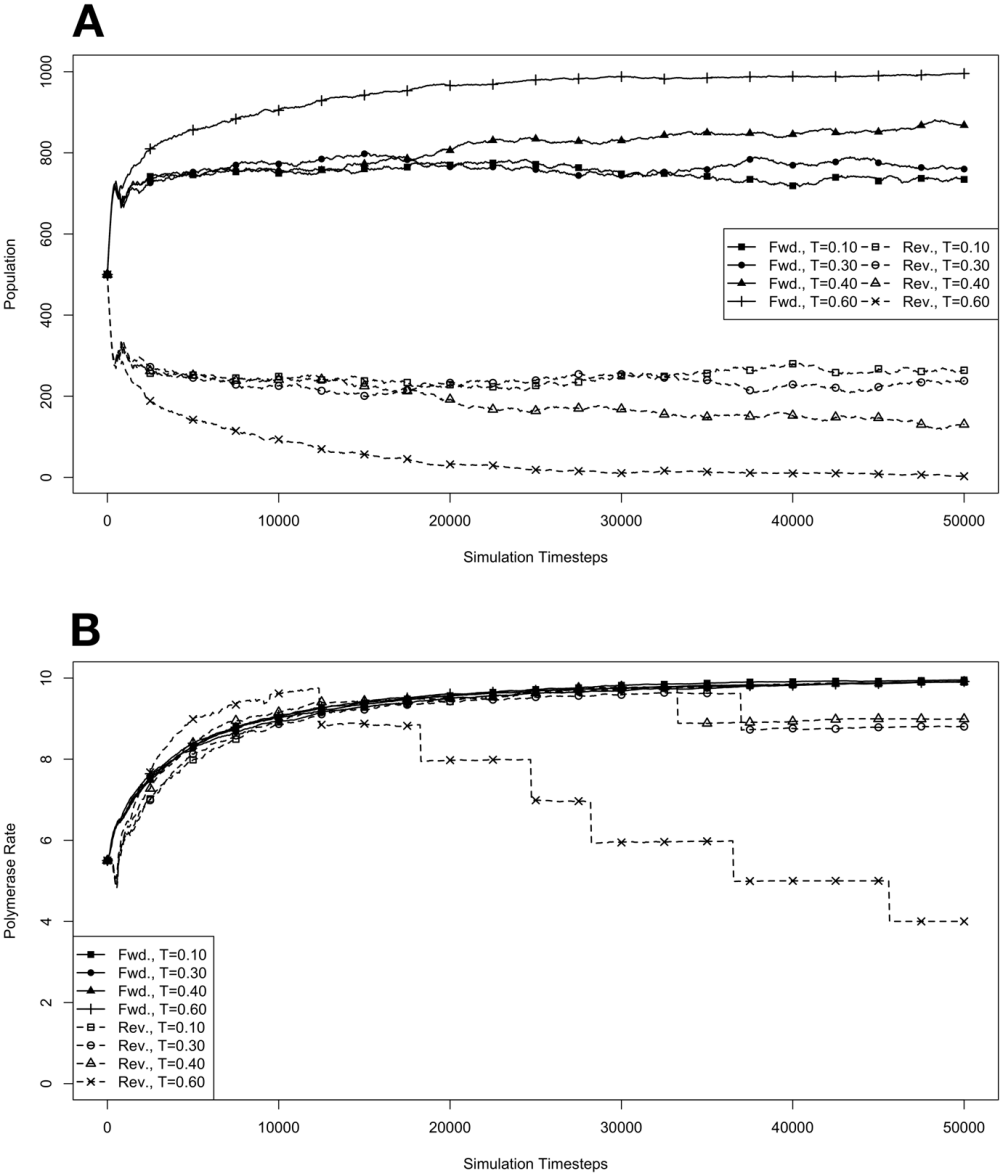
Competition in an environment at maximum capacity, with no mutations. Environments, at simulation temperatures of 0.10, 0.30, 0.40, or 0.60, were seeded with 500 organisms containing forward polymerases and 500 containing reverse, both with a 5.5 average polymerase rate. **A**. Population size of model organisms as a function of simulation time. **B**. Evolution of the average polymerase rate for the organisms in each environment as a function of simulation time. In each case, solid lines are used to indicate environments with forward polymerizing organisms and dashed lines are for reverse polymerizing organisms. For every simulation mutations were disallowed. Different temperatures are indicated with different data markers as indicated in the figure legend, and are expressed in units of 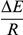.

To further investigate how organisms with forward polymerases fared in competition with organisms with reverse polymerases, specifically to see if an equilibrium was possible between the two varieties, we performed a number of simulations starting with an environment already at its maximum capacity. This was done by seeding each environment with 500 organisms containing forward polymerases and 500 containing reverse. For each variety, there were 50 organisms seeded with each of the 10 possible polymerase rates. The results of these simulations are presented in figure 5. As before, these conditions were repeated with mutations disallowed, and those results are presented in figure 6.

**Figure 7:**
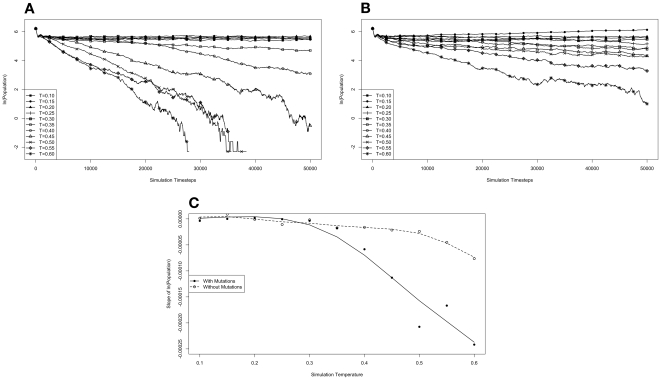
Competition in an environment at maximum capacity at various temperatures. Simulations were carried out with environments at temperatures ranging from 0.10 to 0.60 in 0.05 increments. In each simulation, the environment was seeded at full capacity with 500 organisms containing forward polymerases and 500 containing reverse. For each variety there were 50 organisms with each of the possible polymerase rates, giving an average rate of 5.5 In **A** and **B** the natural log of the population of organisms containing reverse polymerases is plotted as a function of simulation time. **A** is the data from simulations where mutation was allowed, and **B** is from simulations where mutations were not permitted. A plot of the slopes of a least squares regression line for the data in each simulations is plotted as a function of simulation temperature. Data from simulations with mutation is plotted as the solid line with data from the no mutation simulations plotted as a dashed line.

We noted that the results of this last experiment seemed to indicate that, when mutations were allowed, their might be a temperature regime in which there is a transition between an equilibrium of organisms containing forward and reverse polymerases and a complete dominance by organisms containing the forward polymerase. To get a more detailed picture of this transition temperature, we repeated the simulations with full environments at temperatures from 0.10 to 0.60 in 0.05 increments. Figure 7 shows the results of these simulations.

## Discussion

### Effect of Temperature on Evolution

In constructing the model presented here, we were attempting to evaluate the plausibility of the hypothesis that all life contains nucleotide polymerases which proceed in a 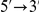 direction due to an evolutionary advantage of this mechanism versus the reverse 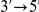 direction. In order to address this question while still remaining biologically relevant, it was necessary to account for a pair of temperature dependent chemical processes: spontaneous hydrolysis of nucleotide triphosphates and inclusion error rate. Unfortunately, due to the simplified nature of the model it was impossible to correlate, with any confidence, a model temperature to a real temperature. The next best thing we could do, then, was to investigate the effect that temperature would have on the outcome of the competition in our model system.

First, to validate the kinetics of the model we carried out a number of simulations in which each sort of organism, those containing the 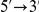 (forward) polymerase and those containing the 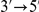 (reverse) polymerase, were allowed to grow from a small starting population in the absence of competition (fig. 1**A**). These experiments revealed that the temperature of the environment had a gradual impact on the growth rate of the model organisms, with the greatest inhibition on growth rate occurring at the highest temperatures investigated. At these higher temperatures, the effect on growth appeared to be greater on organisms containing the reverse polymerase than on those containing the forward polymerase. This indicates that the disadvantages inherent in polymerizing nucleic acids in a 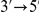 direction become more exaggerated as the temperature increases.

Another way to analyze these experiments is to look at the way that the rate of the nucleotide polymerases evolves at each temperature. Generally, we can state that tendency toward a faster polymerase indicates that the predominant evolutionary pressure is on reproduction rate, while a tendency toward slower polymerases indicates an increased importance of reducing the mutation rate. This stems from the fact that a faster polymerase will have a greater error rate. With this in mind, figure 1**B** shows that, as the temperature of the simulation increases, the average polymerase rate decreases indicating the increasing importance of avoiding errors. This is true up to simulation temperatures of 0.40, but at a simulation temperature of 0.60 the trend reverses indicating a switch back toward reproduction rate as the primary evolutionary pressure.

**Figure 8:**
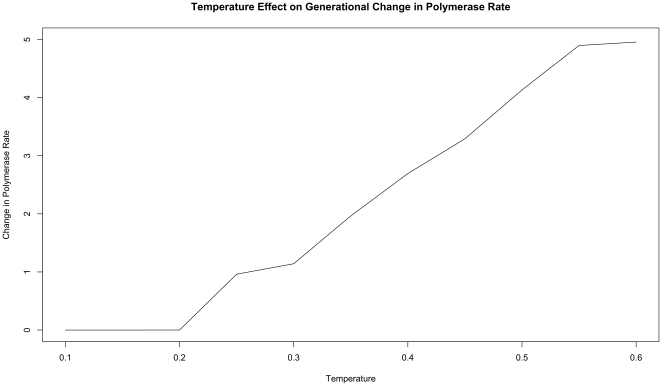
Generational change in polymerase rate as a function of temperature. The organisms from the systems plotted in 1 were analyzed for the difference between the rate of their polymerase and the rate of their parent's polymerase. This difference is plotted for the various different temperatures simulated. The maximal difference we would expect to see (i.e. in the case that inheritance was purely random) would be 5.

This switch most likely represents a saturation of the effect that mutation can have on the organisms. That is, in our model system an increasing mutation rate decreases the fidelity with which each generation can pass along its traits (in this case just polymerase rate) to subsequent generations. We expect that, at some high mutation rate, this fidelity drops significantly enough that the polymerase rate inherited from one generation to the next is not significantly distinguishable from a randomly assigned value. When the temperature of the simulation gets high enough, the error rate due to the thermal component will overwhelm the error rate due to polymerase rate, and reach this critical value. At this point the selective pressure against faster polymerases resulting from the need to preserve generation to generation fidelity will, in effect, vanish. In our system, this leaves growth rate as the single remaining selective pressure, explaining the reversal of the trend of polymerase rate evolution at high simulation temperatures. Indeed, as can be seen in figure 8, at simulation temperatures of 0.55 and 0.60, the average change in polymerase rate from one generation to the next is nearly 5, the maximum that would be expected if polymerase rates were inherited at random.

One interesting result from this first experiment that was not completely anticipated was that the polymerase rate of the forward and reverse polymerizing organisms would be so similar over a wide range of simulation temperatures. We expected that reverse polymerizing organisms, with the increased penalty for a spontaneous hydrolysis event, would have a greater selective pressure to evolve a faster polymerase holding all else constant. This seems to not be the case, though, as the average polymerase rate of the forward and reverse polymerizing organisms at all but the highest temperature are indistinguishable. The only conclusion we can draw from this observation is that the selective pressure on mutation rate is sufficiently rigid enough to make the spontaneous hydrolysis penalty a negligible influence on the evolution of polymerase rate. This conclusion is further backed up by evidence from the simulations with no mutation allowed, as described below.

The next set of experiments was intended to look at how forward and reverse polymerizing organisms would fare in competition with each other at various temperatures. We again started each simulation with a small seed population and allowed these populations to grow in an exponential fashion. As we expected based on the similarity of growth rates for the forward and reverse polymerizing organisms growing in isolation, both forms grew rapidly until the environment was at capacity with approximately half of the population belonging to each form. At this point, competition kicks in and at simulation temperatures of 0.1 an equilibrium between the two forms is established. At a simulation temperature of 0.3, it is unclear in the duration of the simulation run whether an equilibrium might eventually be established or whether the reverse polymerizing organisms would eventually be outcompeted. At the higher simulation temperatures of 0.4 and 0.6, the reverse polymerizing organisms are outcompeted by the forward polymerizing forms, however at the temperature of 0.4 this is a true out-competition, where the reverse polymerizing form grew to occupy nearly half the population before being eradicated. At a simulation temperature of 0.6, the reverse polymerizing organisms are never able to establish themselves.

Looking at the evolution of the polymerase rates of the forward and reverse polymerizing organisms, it is apparent that the selective pressure on polymerase rate is greater when there is competition involved at low temperatures. That is, at a simulation temperatures of 0.3 and 0.4, the steady-state polymerase rate under competition is not appreciably different than under the no competition growth case, but at a simulation temperature of 0.1 the steady-state polymerase rate under competition is elevated as compared to the average polymerase rate for both forward and reverse polymerizing organisms growing in isolation. From this we can conclude that rapid growth takes on an increased importance when competition with an alternative strategy is involved.

Because the reverse polymerizing organisms were not able to grow significantly at a temperature of 0.6 under competition, and because of the ambiguity of the eventual fate of the reverse polymerizing organisms at a temperature of 0.3, we were curious what might happen if both forms started off at a significantly larger population size. Specifically, we were interested to see if forward polymerizing organisms represented an evolutionarily stable strategy at these temperatures ([8], chap. 4). In order to investigate this question, we carried out simulations starting with completely full environments split evenly between forward and reverse polymerizing organisms at various temperatures.

The results from these simulations (fig. 5) indicate that the ultimate success of the forward and reverse strategies in competition with each other when starting from half of an environment at capacity is similar to that during exponential growth, though the dynamics are interestingly different. In general, we can conclude that forward and reverse polymerizing organisms will establish an equilibrium at lower temperatures and that forward polymerizing organisms are dominant at higher temperatures. That is, at sufficiently high enough temperatures, polymerizing nucleotides in a 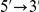 direction appears to be an evolutionarily stable strategy.

Also, the steady state polymerase rates reached at each temperature are the same as for the exponential growth case above. The simulations starting with a full population differ from those starting with exponentially growing populations in how the steady state is reached. Arrival at the steady state polymerase rate occurs much earlier in the case of exponential growth. This reflects the fact that in a mostly empty environment it is easier for the best fit organisms to rapidly dominate a population. In a population already at capacity, this rebalancing of traits can only occur through attrition, which is a more gradual process. Interestingly, the rate at which the steady state polymerase rate is achieved appears to impact the distribution of the equilibrium population. At a simulation temperature of 0.1, the equilibrium population in the exponential growth case is evenly divided between forward and reverse polymerizing organisms, but in the full environment case the split is closer to 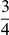 forward polymerizing organisms and 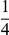 reverse. We believe that this difference may point toward the existence of different domains of competition. That is, it may be possible that the fitness of the model organisms is impacted by the population relative to the environment's carrying capacity. Since there are two fitness components encoded in the polymerases of our model organisms, namely proliferation rate and mutation rate, what we may be observing is the existence of multiple fitness equilibria between the forward and reverse polymerizing organisms. Future models wherein these fitness components could be decoupled might help resolve this possibility.

### Effect of Mutation on Evolution

To better understand the role that mutation and the introduction of random variation from generation to generation might have on the results of our simulations, we modified the model system to remove the possibility for mutation. With this modification, the average polymerase rate can still evolve, but only through selection of individuals. Also, since the only evolutionary pressure acting on the model organisms is reproduction rate, we would expect that all individuals should converge on the maximum polymerase rate. Indeed, this is precisely the result we observe (fig. 2**B**). With the selective pressure of mutation removed, we can also directly observe the impact of the penalty on reverse polymerizing organisms due to spontaneous hydrolysis of nucleotide triphosphates. In figure 2**B**, we can see that at simulation temperatures of 0.4 and 0.6 the reverse polymerizing organisms evolve to faster polymerase rates sooner than their forward polymerizing counterparts.

We also note that the exponential growth of both the forward and reverse polymerizing organisms is essentially identical at all temperatures except for 0.6 (fig. 2**A**). Reverse polymerizing organisms at a simulation temperature of 0.6 are the only group to deviate. This is due to the excessive penalty paid by a reverse polymerase at this high temperature. Moreover, the reverse polymerizing organisms at a simulation temperature of 0.4 appear to grow with identical kinetics to the forward polymerizing organisms at this temperature, showing that the penalty of a reverse polymerizing strategy can be compensated for by evolving a faster polymerase (as seen in fig. 2**B**).

Following this initial result, we then repeated the prior experiments starting with small populations and starting with a full environment, but with mutations disallowed (figures 4 & 6, respectively). When we start with small populations we see, as before, that polymerase rate rapidly evolves to its equilibrium value (fig. 4**B**). The difference here is that without mutation this equilibrium value at all temperatures is the maximum polymerase rate allowed. At the highest temperature of 0.6, the reverse polymerizing organisms are unable to establish themselves the same as when mutations were allowed, implying that this inability to compete with forward polymerizing organisms during exponential growth is a consequence of the spontaneous hydrolysis penalty and is not affected by the presence or absence of mutations in the system.

Paying attention to the way that the competition between forward and reverse polymerizing organisms resolves itself at a simulation temperature of 0.4 when starting with a small population (fig. 4**A**), its not entirely clear whether an equilibrium between these forms can be established. If we look for the establishment of an equilibrium when starting with a full environment at the same temperature (fig. 6**A**), it does appear that the forward polymerizing organisms are outcompeting the reverse polymerizing organisms, but the rate at which this take over happens is definitely slower than when mutations are allowed (fig. 5**A**).

This leaves a question of whether allowing mutations, which affect the generation to generation inheritance of polymerase rate, allow for a more rapid resolution of the more dominant strategy, or whether the addition of mutation to a reverse polymerase strategy causes this to be a loosing strategy when it otherwise might not be. We noticed that the decline in population of reverse polymerizing organisms when starting from a full environment is roughly exponential, so we plotted the natural log of this number against simulation time in figures 7, **A** & **B** for simulations where mutations were allowed in the system or where they were prohibited, respectively. These plots represent the rate of population change in each simulation. By calculating the least squares regressions of these rates and plotting the slope of these regressions versus the temperature (fig. 7**C**), we can see clearly that there is an inflection point for both the mutation allowed and the mutation disallowed situations, but that it occurs at different temperatures.

When an equilibrium between forward and reverse polymerizing organisms is established, we expect the rate of population change to not be significantly different from zero. At low simulation temperatures, this is indeed the case. The inflection of the plots in figure 7**C** represents the point at which forward and reverse polymerizing organisms go from being able to establish an equilibrium to a situation where the forward polymerizing organisms are an evolutionarily stable strategy, and the reverse polymerizing organisms will eventually be eradicated from the environment. That this inflection occurs at different temperatures with or without mutations allowed indicates that there is a range of simulation temperatures where mutation does, in fact, make the difference between a forward polymerizing strategy being evolutionarily stable versus merely being the dominant strategy.

### Founder Effect or Evolution?

Finally, returning to our original question of whether the present day situation that finds all organisms polymerizing nucleotide polymers in the same 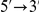 direction is the result of a founder event or a consequence of evolution toward a best fit trait, unfortunately the evidence is equivocal. Here we have presented a model system that could potentially explain how evolution might account for the rise of a single polymerase strategy.

At the same time, we have shown that this strategy can coexist with the alternative under the right conditions. That is, at a high temperature it seems clear that polymerizing nucleotides 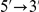 is clearly favored, and would be selected for fairly rapidly. At lower temperatures evolution leads to a predominance of one form, which would make a founder event resulting in a 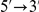 polymerase more likely than the reverse, but evolution alone cannot explain the convergence to a single polymerase strategy.

Really, our inability to link simulation temperature to an actual environmental temperature with any great confidence makes it so that we must leave it at this. We can state that life originating in proximity to a deep sea hydrothermal vent would be more likely to arrive at a single strategy through evolution than life originating in Darwin's “warm puddle”, though it is possible that both environments would be either above or below the temperature at which evolution leads to a single polymerase form. While this is the most we can conclude from the evidence presented here, combined with other evidence that the machinery involved in DNA polymerization evolved at least twice independently [9], the weight of evidence favors the explanation that evolutionary competition was the driving factor in determining the modern strategy of nucleotide synthesis.

### Significance of Results

Though the evidence presented may not provide conclusive support for or against our original hypothesis, the system constructed and the results obtained from it do present many lessons and interesting leads for future investigations. This is the first system that we are aware of that combines both modeling of the “evolutionary short-loop”, where mutation alters the replication mechanism which controls the rate of mutation, and simplified real-world thermodynamics of the biochemical processes involved in life. For this reason, this is also the first time we can look at the direct impact that an environmental factor such as temperature has on evolving systems. This is important for the development of theoretical approaches to evolution which depend on interactions between organisms and their environments.

Finally, the model we constructed allowed us to probe the consequences of mutation on evolution. The role of mutations in evolution has long been debated. On one hand, mutations provide variations which are the fodder of natural selection. On the other hand, mutations reduce the information content passed from generation to generation, thereby reducing the efficiency of selection. Our results indicate that the role of mutations in evolution may be complicated by the nature of the evolving system. At low simulation temperatures, mutations appeared to have little to no effect on evolution and competition between forms. In a middle range of simulation temperatures, mutations are the difference between a strategy being able to successful coexist with another and that strategy being a loosing strategy that is eventually eliminated. Finally, at the highest simulation temperatures, the only difference introduced by mutations is a difference in the rate at which the successful strategy is able to outcompete the alternative. This insight into the role that mutations play will surely be invaluable for future investigations.

## Methods

### Model Design

The model used to carry out all of the simulations described was constructed as a series of nested objects in the Ruby programming language and run on an Apple Mac Pro with Ruby 1.9.1 compiled from the C Ruby source. In the model, the outermost object was the environment which was populated with organism objects as described in the text. After each round of simulation, a determination was made as to whether or not to remove an organism from the environment based on a probability described by the equation:
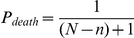
where 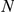 is the environment's carrying capacity, 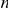 is the number of organisms currently in the environment, and 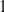 is added so that the probability of at least one organism being culled from the environment when the carrying capacity is reached is 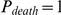. If one organism is removed, then this probability is calculated again to decide on removing a second organism. This process is repeated until no organism is removed.

Organisms in the simulation all started with a random percentage of their genome already synthesized to avoid synchronization artifacts that arise when all organisms begin synthesizing a new genome simultaneously. Organisms were modeled as finite state machines that were either in a polymerizing state or a dividing state. In the polymerizing state the organism's polymerase was allowed to add nucleotides to the nascent genome. When the genome was completed, a state change occurred and the organism was placed in the dividing state, where it would remain until it was able to add its daughter organism to the environment. Upon successful division, the state returned to polymerizing, and the polymerase was allowed to begin synthesis of another new genome.

Polymerases in the simulation were endowed with a polymerase rate from 1 to 10. This rate determined the maximum number of nucleotides that a polymerase could add to a growing genome during each simulation time step. During each time step, each polymerase entered an addition loop. Before each addition in this loop, the polymerase checked for a spontaneous hydrolysis event. For the forward polymerases, this was based on the Boltzmann distribution:
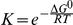
where 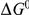 is the standard free energy of the hydrolysis reaction. To simplify the simulation calculations, the entire 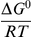 term is expressed as a generic simulation temperature 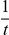. For the reverse polymerases, this distribution was adjusted by a factor determined by the polymerase rate:
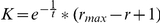
where 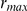 is the maximum polymerase rate and 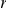 is the rate of the polymerase doing the addition. This extra factor accounts for the fact that a slower reverse polymerase will give each terminal phosphate group on the growing chain a longer period of time before addition of the next nucleotide monomer during which a spontaneous hydrolysis might occur. In the case of a forward polymerase, the calculated probability was compared against a randomly generated value. In the case that this comparison indicated that a hydrolysis had occurred, that specific addition was skipped, and the addition loop proceeded to the next iteration. In the case of a reverse polymerase, when the same sort of comparison indicated a spontaneous hydrolysis, the addition loop was halted until the next simulation time step.

At the point of nucleotide addition, the probability that an erroneous nucleotide would be included was calculated based on two factors. First, the hydrogen bond interaction that allows discrimination between Watson-Crick base-pairing and a variety of mismatches was modeled using the same sort of Boltzmann distribution as used for the spontaneous hydrolysis condition. However, since the 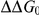 of a correct versus incorrect base pairing is twice that of the spontaneous hydrolysis reaction [10], [11], the hydrogen bond portion of the erroneous inclusion calculation is made using the formula:
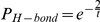
where 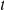 is the simulation temperature. The second factor in determining whether the included nucleotide is erroneous or not is related to polymerase rate. Prior studies have revealed a relatively complex link between polymerase rate and error rate for nucleotide polymerases [12]. The essence of this link is that there is an additional geometric constraint on erroneous nucleotide inclusions and that, in order to polymerize faster, polymerases must relax this constraint. Therefore, to model this relationship as simply as possible, we consider polymerase rate as the flux of a nucleic acid polymer through a cylindrical tube, and the geometrical constraint as being directly correlated to the radius of this tube. This gives us a total erroneous inclusion probability calculated by:
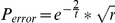
where 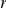 is the polymerase rate.

The genome for each organism in the simulation was a simple object which tracked its length, the number of nucleotides added to the nascent copy being made of itself, the number of erroneous nucleotide inclusions during synthesis of the copy, and the polymerase rate and directionality for which it coded. When the number of nucleotides added to the copy equals the length, the genome signals the organism to switch to the dividing state. During division, the genome sets the polymerase rate and directionality of the newly synthesized copy. The directionality is set the same as the parent. The polymerase rate will deviate from that of the parent genome based on the number of errors introduced during replication. The algorithm used to determine the generational difference in rate is:
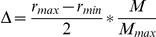
where 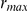 and 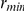 are the maximum and minimum possible rates, respectively, 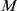 is the number of errors made during replication, taken as a fraction of total genome length, and 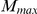 is the maximum tolerated fraction of errors which was set at 0.34 in our model. The calculated difference was then applied to the polymerase rate encoded in the mother genome in such a way that the polymerase rate encoded by the daughter genome remains in the set 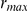 to 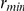 range. If the difference could be added or subtracted and still remain within this range, then addition or subtraction was chosen at random.

### Data Analysis

Each simulation described was run 10 times, and for each simulation time step the arithmetic mean value for the 10 runs was taken for further analysis. Simulations of the growth of organisms containing forward or reverse polymerases in the absence of competition were run for 5000 simulation time steps with the statistics for the simulation populations collected every 5 time steps. For all of the remaining scenarios investigated, simulations were run for 50000 simulation time steps with the statistics for the simulation populations collected every 50 time steps. All figures were prepared using the R statistics package [13]. Plots of population versus time and average polymerase rate versus time were simple line plots of the full data set with data markers every 50 samples. To plot the slope of the log of reverse polymerizing organism population at various temperatures, a simple least squares regression was first calculated for the plot of 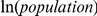 vs time, then the slopes of these regressions were plotted versus temperature, and a smoothed spline regression was plotted for the data.
